# Tuberculosis drug discovery in the CRISPR era

**DOI:** 10.1371/journal.ppat.1007975

**Published:** 2019-09-19

**Authors:** Jeremy Rock

**Affiliations:** Laboratory of Host-Pathogen Biology, The Rockefeller University, New York, New York, United States of America; Nanyang Technological University, SINGAPORE

## Abstract

Stewart Cole and colleagues determined the complete genome sequence of *Mycobacterium tuberculosis* (Mtb), the etiological agent of tuberculosis (TB), in 1998 [1]. This was a landmark achievement that heralded a new age in TB drug discovery. With the genome sequence in hand, drug discoverers suddenly had thousands of new potential targets to explore. But the excitement has since faded [2]. It is unquestioned that genomics has transformed our understanding of the biology of this pathogen. However, the expectation that the Mtb genome sequence would rapidly lead to new therapeutic interventions remains unfulfilled [3]. One of the (many) reasons for this unrealized potential is that our tools to systematically interrogate the Mtb genome and its drug targets—so-called functional genomics—have been limited. In this Pearl, I argue that the recent development of robust CRISPR-based genetics in Mtb [4] overcomes many prior limitations and holds the potential to close the gap between genomics and TB drug discovery.

## New antibiotics are needed to treat TB

Despite the availability of tuberculocidal antibiotics since the 1940s, over one-quarter of the world remains infected, and approximately 1.6 million people die of TB every year [5]. Public health efforts to restrain the TB pandemic are limited by many factors, not least of which includes the inadequacies of current TB chemotherapy. Drug-sensitive TB is treated with combination chemotherapy for six months under so-called directly observed therapy (DOT)—an enormous burden on already strained public health infrastructures. Prolonged chemotherapy and adverse events limit patient compliance, which, in turn, increases the likelihood of continued Mtb transmission as well as the evolution of drug resistance. Drug-resistant TB is now common in many parts of the world and is very difficult to treat [5]. For these reasons, a fundamental goal of the TB field is to develop a regimen of new drugs with novel targets and mechanisms of action (MOA) capable of treating both drug-sensitive and drug-resistant TB in weeks rather than months or years.

## Discovering new TB antibiotics—Two complementary strategies

There are two complementary approaches to discover new TB antibiotics: a genes-to-drugs model (target-based) and a drugs-to-genes model (drug-based). Target-based drug discovery was enabled by the sequencing of the Mtb genome and annotation of thousands of new potential drug targets. In a target-based approach, a drug target is selected, purified, and used to identify small molecules that modulate in vitro target function, typically assaying for inhibition. Target-driven approaches have a significant advantage in that new targets can be rationally prioritized based on attractive characteristics, for example, new targets that are not subject to pre-existing drug resistance, have a structurally exploitable active site, are localized on an extracellular surface to facilitate antibiotic access, and numerous other reasons. While target-based approaches have yielded profound advances in our ability to treat some viral diseases and cancers [6], the results for antibacterials have been underwhelming [2]. Target-based approaches have yet to produce a single new drug used to treat TB [7]. While the reasons for the failure of target-based approaches are multifactorial, oft-cited examples include insufficient chemical diversity in screening libraries (note that antibiotics frequently do not follow Lipinski’s rule of five) and the inability of drug-like small molecules to penetrate bacterial cell walls, evade efflux, and avoid xenobiotic metabolism [7].

Recognizing the challenge of target-based approaches, the TB field has largely returned to phenotypic screens for drug discovery. Here, large compound libraries are screened to identify whole-cell active inhibitors of Mtb growth. Such phenotypic screens have been extremely productive and delivered all drugs currently used to treat TB, from the discovery of streptomycin in 1944 to the approval of bedaquiline in 2012 [7]. However, phenotypic screens are not without their own limitations. These include 1) the frequent rediscovery of compounds targeting a limited set of pathways and so-called “promiscuous” Mtb targets, such as *mmpL3* and *dprE1* [3]; 2) the molecular target and MOA must be identified for hit compounds to progress, and discovering MOA is frequently nontrivial [7]; and 3) the differences between growth conditions provided during in vitro screening and those found during in vivo infection can discover potent in vitro growth inhibitors with MOAs that are irrelevant during infection [8].

## CRISPR interference as an enabling technology for TB antibiotic discovery

Given the limitations of both target- and small-molecule–centric strategies, future TB antibiotic discovery efforts will continue to rely on the complementary use of both approaches. Target-based approaches hold the promise of expanding drug target space to include the most biologically attractive targets but have an unacceptably high target attrition rate; whole-cell phenotypic screens can be productive but often suffer from a restricted target space and lack of up-front knowledge of hit MOA. There are numerous opportunities—encompassing chemical, biological, bioinformatic, and technical advances—to improve TB antibiotic discovery. Given the scope of this Pearl, I focus here on the application of new CRISPR-based tools in Mtb [4] to address three structural problems facing TB antibiotic discovery.

### CRISPR interference

**Fig 1:**
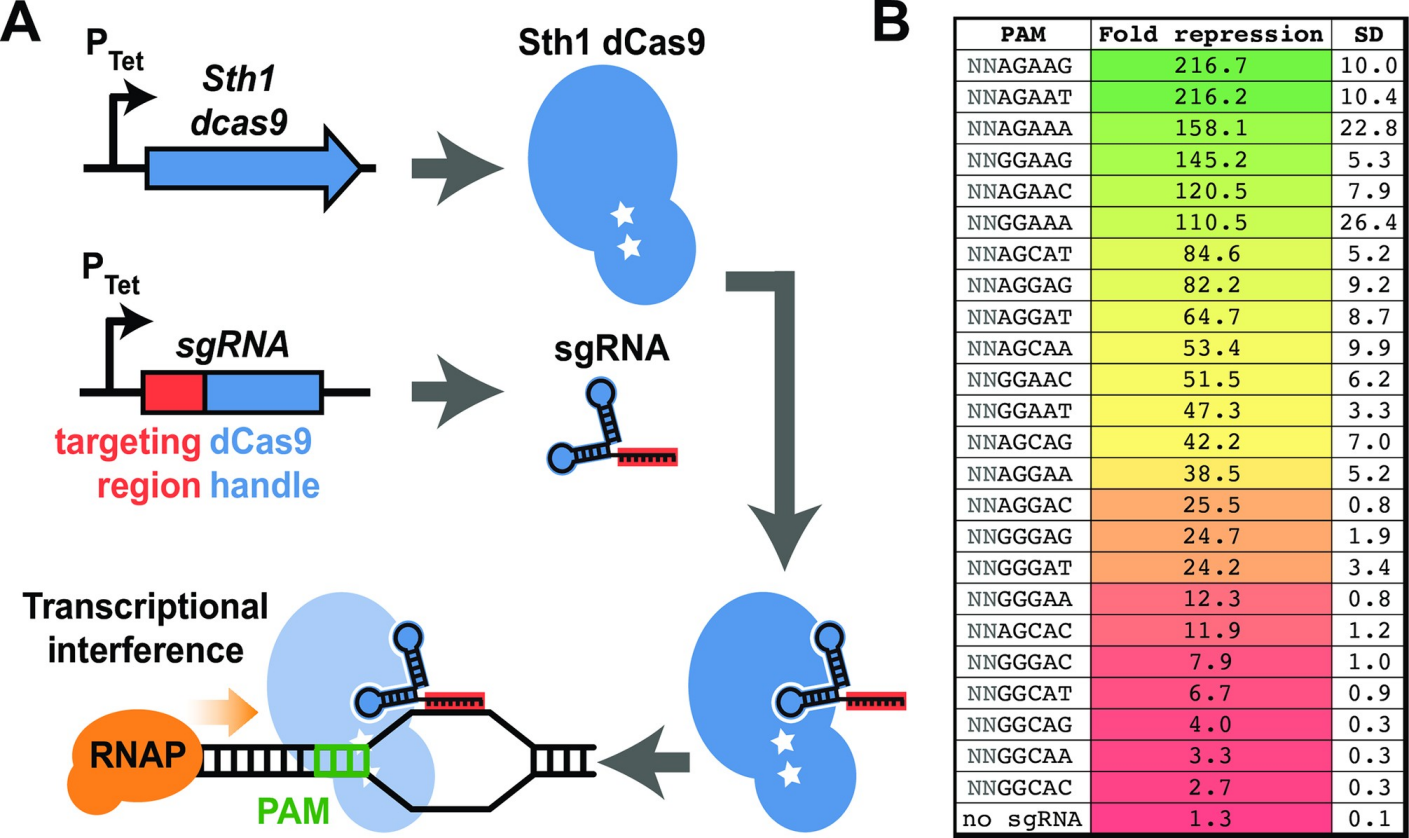
Bacterial CRISPRi. **(A)** Schematic of the optimized Mtb CRISPRi mechanism. Addition of the small-molecule inducer anhydrotetracycline or doxycycline induces Sth1 dCas9 and sgRNA expression, resulting in transcriptional silencing of the target gene. Note that CRISPRi can be used to block transcription initiation or elongation by binding to the target gene promoter or open reading frame, respectively. See the main text for details. **(B)** The magnitude of target gene knockdown can be tuned by utilizing divergent PAMs with Sth1 CRISPRi. Using an identical sgRNA and *Renilla* luciferase target, different PAM sequences (first column) were tested for their ability to mediate transcriptional silencing by Sth1 CRISPRi (second column) in the model mycobacterium *Mycobacterium smegmatis*. Increasing the divergence from the consensus 5’-NNAGAAW PAM progressively reduces the level of transcriptional silencing achieved. Data reproduced from [4]. CRISPRi, CRISPR interference; dCas9, nuclease-dead Cas9; PAM, Protospacer Adjacent Motif; P_Tet_, Tet repressor regulated promoter; RNAP, RNA polymerase; SD, standard deviation; sgRNA, single guide RNA, Sth1, *Streptococcus thermophilus* CRISPR1.

We recently developed an optimized CRISPR interference (CRISPRi) system for targeted gene silencing in Mtb (**Fig 1**) [4]. Unlike most other CRISPRi applications, which utilize a Cas9 enzyme derived from *Streptococcus pyogenes* [9–11], we found a Cas9 enzyme derived from *S*. *thermophilus* to have superior performance characteristics (magnitude of target gene knockdown and reduced toxicity) in *Mycobacterium smegmatis* [4]. In this system, the protein dCas9 (with two mutations that disable nuclease activity, thus “dead” or dCas9) is guided to the target gene by a chimeric RNA called a single guide RNA (sgRNA) [12]. Targeting specificity is determined both by base pairing of the sgRNA and target DNA as well as a short DNA motif (protospacer adjacent motif [PAM]) within the target DNA sequence. The PAM is an approximately 2–8 base pair sequence located immediately downstream of the sgRNA target sequence [13]. PAM recognition is an obligate first step for dCas9 binding—recognition of the PAM by dCas9 destabilizes the adjacent DNA duplex, thereby allowing interrogation of the DNA target by the sgRNA [14]. Binding of the dCas9–sgRNA complex to the target gene results in transcriptional interference by blocking RNA polymerase promoter access or transcription elongation [9,10].

The Mtb CRISPRi system described previously [4] was further engineered to be inducible by 2 alternative tetracycline small molecules (anhydrotetracycline or doxycycline), thereby allowing the facile manipulation of Mtb drug targets, be they essential or nonessential for in vitro growth. The efficient cellular and tissue penetration of doxycycline (Veronique Dartois, personal communication) should allow CRISPRi-mediated control of the Mtb transcriptome in numerous experimental settings, including axenic in vitro culture, ex vivo Mtb-infected macrophages, and during in vivo animal infection models [15]. An additional strength of this Sth1 dCas9 system is that the magnitude of target gene silencing is tunable, either by varying targeted PAM “strength” [4] or varying the length of the sgRNA targeting sequence [9], thereby allowing rheostat-like control of target gene production spanning two orders of magnitude [4]. Tunability enables the hypomorph, or partial silencing of target gene production, thus allowing the study of interactions (chemical and genetic) between otherwise in vitro essential genes [4]. Lastly, CRISPRi is scalable. With advances in array-based synthesis, generating large pools of unique sgRNA targeting sequences (>90,000) is fast and inexpensive. These sgRNAs can be cloned as pools to build high-diversity CRISPRi libraries targeting all or nearly all Mtb genes. Combined, these unique features set the stage to develop CRISPRi as a powerful new method for functional genomics in Mtb. Next, I discuss how this tool might improve TB antibiotic discovery.

#### Question 1: How can we bias target-based drug discovery towards targets with higher chances of success?

Most clinically relevant antibiotics target a very limited set of biological pathways. The evolution of drug resistance to existing antibiotics is making the targeting of these pathways increasingly ineffective. Thus, it is clear that TB antibiotic discovery needs to expand target space.

An “ideal” new Mtb drug target would satisfy numerous criteria. Such criteria would include the following: a) a novel biological process not subject to pre-existing drug resistance; b) druggability (although the definition of druggability will likely expand over time, as beautifully exemplified with proteolysis targeting chimeras [PROTACs] [16]); c) be sufficiently diverged from any human homolog to allow specific targeting; and d) essentiality across the diverse physiologic states in which Mtb exists during infection. The importance of validating the in vivo essentiality of a target should not be underestimated [8]. Here, genome-scale CRISPRi presents a complementary approach to high-density transposon mutagenesis studies to define essential Mtb genes [17]. Given the ability to control Mtb CRISPRi with small-molecule inducers, it will be possible to systematically define those bacterial genes essential for in vitro growth, ex vivo macrophage infection, and in vivo animal model infection. Further, given the efficacy of doxycycline in numerous mammalian species (Veronique Dartois, personal communication), it should be possible to profile Mtb gene essentiality across diverse animal models with spectrums of disease and pathologies similar to those seen in humans. Such profiling may thus allow the comprehensive identification of Mtb genes essential for bacterial survival in diverse pathological environments—in essence, to genetically validate a “pan” essential Mtb genome.

But essentiality alone may not be a sufficiently compelling reason to prioritize a target for drug discovery. Amongst all essential targets, might there be targets more likely to yield to chemical inhibition? To answer this question, CRISPRi could be used to define target vulnerability [4]. Here, vulnerability is quantitatively defined by the magnitude of protein knockdown necessary to achieve bacterial growth inhibition. Importantly, experiments with six drug targets in the model mycobacterium, *M*. *smegmatis*, suggests that indeed, genes essential for in vitro growth vary widely in their susceptibility in knockdown [18]. One can thus imagine constructing a genome-scale Mtb CRISPRi library composed of sgRNAs of widely varying predicted strengths (and presumably target gene knockdown), as afforded by the tunability of the Sth1 dCas9 system. Using this approach, a vulnerable target would be a gene for which even predicted “weak” sgRNAs result in growth inhibition, whereas an invulnerable target would be one for which only the “strongest” sgRNAs inhibit growth. It seems reasonable to conjecture that vulnerable targets may yield more readily than invulnerable targets to even weakly efficacious compounds. Thus, prioritizing vulnerable targets for target-based screening may tip the scale in favor of the screener and increase campaign success rates.

Should such an approach prove feasible, it may further be advantageous to quantify target vulnerability across diverse Mtb clinical isolates. Growing evidence suggests differential efficacy of some antibiotics against otherwise drug-sensitive Mtb clinical strains [19]. Given the easily portable nature of CRISPRi and the relatively small amount of genetic divergence between any two Mtb clinical isolates (approximately 1,500 SNP distance between two diverged lineages) [20], CRISPRi libraries designed against one Mtb strain should be effective in other strains. CRISPRi profiling in diverse Mtb clinical isolates may thus enable the interrogation of target vulnerability conservation, with conserved, vulnerable targets being prioritized for target-based screening.

Lastly, an emerging drug discovery approach combines prior target selection with whole-cell screening and is known as target-based whole-cell screening [21]. Here, under-expression of a chosen drug target sensitizes cells to on-target or pathway inhibitors and can be used to selectively identify compounds perturbing the chosen target or pathway. Given the relative simplicity of generating target under-expressing strains with CRISPRi, it is easy to imagine the application of CRISPRi to facilitate target-based whole-cell screening for drug targets prioritized based on the credentialing described previously.

#### Question 2: For the hundreds of compounds that we know have antitubercular activity but don’t understand how they work, how can we discover their MOA?

The output of phenotypic screening has significantly outpaced MOA discovery, leading to the identification of hundreds of compounds with known antitubercular activity but undefined targets and/or MOA [22]. Deducing the MOA of whole-cell active compounds can often be challenging, particularly if the compound has more than one target (which is common), if the target is not a protein, or if off-target mutations can mediate drug resistance (e.g., drug influx or efflux) [7]. Knowledge of compound MOA is desirable with respect to the target credentials listed in Question 1, as well as for the pursuit of structure–activity relationship (SAR) studies to develop more “drug-like” compounds. The lack of understanding of compound MOA is a major impediment to the preclinical development of potentially hundreds of promising new compounds to treat TB.

CRISPRi, in combination with orthogonal approaches, could help alleviate this bottleneck. This assertion is based on the well-founded observation that drug MOA can be predicted by identifying bacterial genes whose activity modulates sensitivity to that drug [21,23–26]. It is frequently the case that drug target levels and drug sensitivities correlate. That is, overexpression of a drug target tends to make cells more resistant to an on-target inhibitor, and under-expression of a drug target tends to make cells more sensitive to an on-target inhibitor. This principle has been used successfully from bacterial to human cells to identify the MOA of whole-cell active compounds [27]. In this context, Mtb CRISPRi has the potential to be developed as a novel chemical genomics platform to predict compound MOA. Here, a genome-scale Mtb CRISPRi library composed of both “strong” and “hypomorphic” sgRNAs—i.e., sgRNAs that mediate partial target knockdown but are still compatible with strain viability [4]—could be screened against a diverse panel of antibiotics with well-defined MOAs. This approach should identify unique sgRNAs and genes that deplete or enrich in response to specific antibiotics. Hit genes may include the direct target(s) of the compound as well as “collateral” targets, which are gene products not directly targeted by the compound but that nevertheless modulate cellular sensitivity to that compound. This constellation of CRISPRi sensitizing and resistance-conferring hits would constitute a chemical-genetic fingerprint, or molecular signature, for each drug. A new lead compound could then be profiled with CRISPRi, and the resulting fingerprint compared with the reference profiles to predict compound MOA. Additionally, because most, if not all, potential drug targets could be targeted for under-expression in the genome-scale CRISPRi library, the molecular target(s) of a compound may be directly revealed as sensitizing hits. This approach should thus be powered to discover compounds with previously known MOAs found in the reference compendium as well as novel MOAs, which are arguably of the most clinical interest. The successful application of this approach would define the MOA of whole-cell active but mechanistically poorly defined antituberculars and enable the further development of these promising compounds.

#### Question 3: How can we rationally develop more potent TB drug combinations?

Treating active TB with monotherapy does not work. As seen shortly after the introduction of the first antituberculous drug, streptomycin, treating TB with a single antibiotic selects for drug-resistant TB and results in treatment failure [28]. In addition to preventing drug resistance, combination therapy is also essential to reduce TB treatment time from greater than 1 year to the current standard of care of 6 months. All evidence suggests that, at least for the foreseeable future, TB will be treated with combination chemotherapy. Thus, a critical question facing TB antibiotic discoverers is “how do we rationally develop new drug combinations that are significantly more potent than what we have now?”

The physiological mechanisms that limit the efficacy of current TB therapy are relatively poorly understood. Broadly speaking, there are two nonexclusive explanations, one bacteria-centric and one drug-centric. Tubercle bacilli are thought to exist in diverse physiologic states within the infected host [29]. These states can arise deterministically (e.g., as a response to local immune pressure) or stochastically (e.g., as a result of noise in gene expression). Some of these physiologic states render Mtb tolerant to one or more antibiotics. These drug-tolerant or persistent bacilli, while genetically drug sensitive, nevertheless are capable of surviving otherwise lethal concentrations of antibiotic. Our understanding of the mechanistic basis for drug tolerance and persistence in Mtb is incomplete but may include metabolic quiescence and dormancy [30], the induction of drug efflux pumps (although it should be noted that efflux represents a classical mechanism of drug resistance rather than tolerance) [31], and the pulsatile expression of drug targets and/or activators [32]. The drug-centric rationale for limited chemotherapeutic potency posits that different antibiotics access bacilli residing in distinct pathological lesions with variable efficacy, for example, as a result of distinct physiochemical properties of each antibiotic. Indeed, work from Dartois and colleagues demonstrated that sterilizing drugs like rifampicin and pyrazinamide efficiently penetrate the sites of Mtb infection, whereas the fluoroquinolone moxifloxacin does so more variably [33]. Thus, differential spatial distribution and kinetics of accumulation in TB lesions may create temporal and spatial windows of less complex drug therapy. Note that the biphasic killing kinetics of Mtb observed during therapy in humans, in which bacilli are killed more rapidly early in therapy than later, is typically ascribed to the presence of drug-tolerant bacilli, but this could equally be a result of variable drug penetration [34]. The relative contribution of these two models, bacterial drug tolerance and drug penetration, to limiting the efficacy of current TB drug combinations remains unknown.

CRISPRi presents distinct opportunities to improve combination chemotherapy. First, CRISPRi could be used to define the bacterial determinants of drug tolerance or persistence. In an extension of the work proposed in Question 1, Mtb CRISPRi profiling could be expanded to animal infection models in combination with antibiotic treatment. Such experiments could identify those genes essential for Mtb to enter or maintain a drug-tolerant or persistent physiologic state. These states likely represent a heterogeneous population of cells with diverse mechanisms, so it remains to be seen what genetic programs may be shared by these mechanisms. That said, this knowledge, combined with additional target credentialing (see Question 1), could then be used for target-based drug discovery campaigns to identify novel compounds capable of eliminating drug-tolerant or persistent Mtb, bacterial subpopulations poorly targeted with conventional therapy.

A second way to develop more efficacious therapies may be to leverage drug interactions. It is hypothesized that synergistic antibiotic combinations—in which an antibiotic combination produces a phenotypic effect that is greater than the additive expectation of the component antibiotics—may be an important mechanism to increase the efficacy of combination therapies. The premise for this hypothesis for TB treatment was the discovery of the antibiotic pyrazinamide (PZA). PZA exhibits synergistic interactions with the first-line antituberculars rifampicin and isoniazid, and inclusion of PZA in first-line therapy is critical to reducing treatment time from approximately 1 year to the current standard of care of 6 months [35]. The molecular target(s) of PZA are controversial, and thus, the mechanistic basis for synergy remains unclear, although recent work suggests that PZA potency may at least, in part, derive from its ability to act in difficult-to-sterilize necrotic lesions [36]. One can imagine synergistic antibiotic combinations overcoming bacterial drug tolerance and persistence, for example, by overwhelming or inactivating drug efflux mechanisms. But synergy might also be able to compensate for uneven drug distribution, for example, by increasing the potency of an antibiotic such that even suboptimal drug penetration could nevertheless achieve sufficient concentrations for therapeutic efficacy. Lastly, there is reason to believe that synergistic drug interactions that promote even small increases in drug potency may have a clinical impact. Emerging evidence suggests that very small shifts in drug sensitivity—2-fold increases in minimum inhibitory concentration (MIC)—can predict higher rates of TB relapse [37]. One interpretation of these data is that some subpopulation of bacilli experience antibiotic concentrations very near the therapeutically relevant MIC, such that a small decrease in drug sensitivity is sufficient to survive therapy. Thus, by extension, even small increases in drug potency should improve therapy.

How might CRISPRi-based approaches help identify synergistic drug targets? One approach could be to apply a chemical-genomic strategy similar to that outlined in Question 2. Here, an Mtb CRISPRi library could be selected at sub-MIC drug concentrations to globally identify genes whose inhibition increases the sensitivity of Mtb to a given antibiotic. Such pathways constitute so-called “intrinsic resistance” mechanisms (as opposed to acquired drug resistance) and are innate mechanisms that impart a bacterial species with decreased sensitivity to antibacterial agents. The pharmacologic targeting of these intrinsic resistance mechanisms could provide a rational basis for the development of synergistic antibiotic combinations. New analytic approaches like machine learning could be applied to identify which target combinations are most likely to lead to drug synergies. Critically, the ability to generate hypomorphic knockdown with CRISPRi should enable the discovery of essential cellular processes that contribute to intrinsic resistance. Drugs developed against such synergistic targets would thus be antibiotics in their own right rather than simply adjuvants that would potentiate other antibiotics.

The chemical-genomic approach outlined previously would complement the important existing efforts to directly assess drug interactions against Mtb [38–40]. Relative to the direct identification of drug–drug synergies, CRISPRi has two main advantages. First, genome-scale CRISPRi is inherently multiplexable, allowing the investigation of thousands of drug–gene interactions in pooled experiments. Quantification of drug–drug interactions is experimentally challenging due to the numerical explosion of possible drug combinations, although new methods are beginning to more efficiently explore drug combination space [38,40]. CRISPRi multiplex-ability should also enable the identification of drug–gene interactions in the context of infection. As discussed previously, Mtb CRISPRi profiling could be expanded to animal infection models in combination with antibiotic treatment to identify intrinsic resistance mechanisms operative during infection. This is important as there is no guarantee that drug–drug or drug–gene interactions identified in axenic culture will be relevant during in vivo infection. Second, assaying drug–drug interactions necessarily limits analysis to those few targets for which we have drugs. CRISPRi, in contrast, can profile most if not all potential drug targets, even those for which we do not yet have inhibitors. Following this logic, one can imagine dispensing with drugs altogether and directly assaying for synergistic genetic–genetic interactions across thousands of genetic combinations with combinatorial CRISPRi knockdown. These approaches should generate a comprehensive list of synergistic drug targets, which can then be queried to determine whether the presence of drug–drug synergies might explain the clinical success of new and existing TB drug regimens.

## Caveat—Genetics does not always equal pharmacology

CRISPRi is not without limitations. It is well known that CRISPRi can induce a polar effect—any operonic gene downstream of the dCas9 binding site will be silenced, in addition to the targeted gene [4,9,10]. The fact that genes in an operon typically function in the same biological pathway partially mitigates this problem. To validate predictions, phenotypes discovered with CRISPRi should be genetically complemented and, ideally, confirmed with an orthogonal experimental approach.

It should also be noted that there are numerous reasons why pharmacologic targeting may not necessarily be mimicked by transcriptional silencing of a drug target [41]. First, transcriptional interference mimics the effects of a noncompetitive inhibitor [42], whereas small molecules have a wider variety of biochemical effects on their target(s), from antagonism to agonism. Second, some enzymes have both catalytic and protein scaffolding functions. Whereas a small molecule may selectively inhibit enzymatic but not scaffolding functions, CRISPRi will necessarily inhibit both [41]. Third, network topology surrounding an enzyme can influence relative susceptibility to genetic knockdown relative to pharmacologic inhibition and produce discordant results between these two experimental modalities [42]. Lastly, CRISPRi function necessarily requires the proper expression and activity of dCas9 and the sgRNA, and thus, any genetic targets, drug treatment, or experimental conditions that perturb dCas9 and/or sgRNA functionality will be missed by this approach.

## Concluding remarks

The past 20 years have seen slow progress in the TB drug pipeline. While optimism is warranted, significant challenges remain. The goal of this Pearl was to highlight three of these challenges and propose solutions enabled by emerging CRISPR-based technologies. The application of these new functional genomic tools may yield a more robust TB antibiotic discovery platform, thereby helping to realize the 20-year-old aspiration of the sequencing of the Mtb genome to develop new drugs to control this disease.
